# Fungal Osteomyelitis of the Hip with Septic Arthritis: Case Report

**DOI:** 10.1055/s-0042-1742604

**Published:** 2022-02-15

**Authors:** João Rodolfo Radtke Gonçalves, Karine Emanuele Tres, Laura Serraglio Narciso, Ricardo Corrêa, Rodrigo Duarte Perez

**Affiliations:** 1 Divisão em Cirurgia do Quadril, Instituto de Ortopedia e Traumatologia, Blumenau, SC, Brasil; 2 Departamento de Ortopedia e Traumatologia, Hospital Santa Isabel (HSI), Blumenau, SC, Brasil; 3 Universidade Regional Blumenau (FURB), Blumenau, SC, Brasil; 4 Departamento de Ortopedia da Universidade Regional Blumenau (FURB), Blumenau, SC, Brasil; 5 Departamento de Infectologia, Hospital Santa Isabel (HSI), Blumenau, SC, Brasil

**Keywords:** arthritis, infectious, orthopedic procedures, osteomyelitis

## Abstract

Fungal osteomyelitis, especially associated with septic arthritis, is uncommon in Brazil; therefore, sometimes it is difficult to diagnose and treat it. We report the case of a young patient, with no immunosuppressive risk factor, with osteomyelitis leading to septic arthritis of the hip. The diagnosis was performed after surgical drainage and visualization of *Cryptococcus neoformans* at pathological anatomy. Antifungal treatment resulted in complete remission of the symptoms. Since there is no consensus on the treatment of fungal osteomyelitis, this case report aims to inform orthopedists about the importance of hip arthritis differential diagnosis and the good evolution of clinical treatment after drainage and pathogen isolation.

## Introduction

Fungal osteomyelitis associated with septic arthritis is an uncommon disease with a challenging clinical approach. It results from direct inoculation of organisms during trauma, surgeries, or procedures such as joint injection or aspiration. Another potential, most common cause is hematogenous dissemination in immunosuppressed patients. Immunosuppression due to chemotherapy, corticosteroid treatment, illicit intravenous drug use, broad-spectrum antibiotic therapy, human immunodeficiency virus (HIV) infection, and organ transplantation, among others, increases susceptibility to hematogenous infections and diseases. 1 2 3 4

The hematogenous route often is correlated with pathogenesis due to the greater synovial and bone tissue vascularization and the lack of a basement membrane limiting the entry of organisms into the joint space. 5 Infection from these sites spreads according to pathogen- and host-related factors.

Although *Cryptococcus neoformans* arthritis usually results from fungi isolated in the osseous locomotor system, literature reports are scarce. 6 7 In fact, the prevalence of fungal arthritis was thought to increase over the years due to the higher numbers of immunosuppressed subjects, the patients most affected by systemic fungal diseases. About half of the patients with cryptococcal arthritis have some predisposing factor, especially knee monoarthritis. However, there are reports on oligoarthritis and polyarthritis in other joints, including the elbow, ankle, sacroiliac joint, and sternoclavicular joint. 8 The initial treatment of fungal infection consists in surgical cleaning and antifungal agents; inadequate management can induce locoregional invasion, resulting in death. 7

Fungal osteomyelitis of the hip is a rare occurrence, motivating this description. We report a rare case of a young woman diagnosed with fungal osteomyelitis in the right femoral neck associated with septic arthritis.

## Case Report

A 20-year-old Caucasian female reported pain in the right hip region for approximately 30 days. Initially, she complained of pain when walking or sitting down for a long time in addition to chills and low-grade fever (37.5 C) during the night. She also reported occasional non-steroidal antiinflammatory drugs use and direct contact with pigeon droppings in rural areas. She denied associated diseases.

On physical examination, the patient presented a limp gait, internal rotation of the right hip limited to 15 degrees, C sign, and a positive flexion, adduction, and internal rotation (FADDIR) test.

**Fig. 1:**
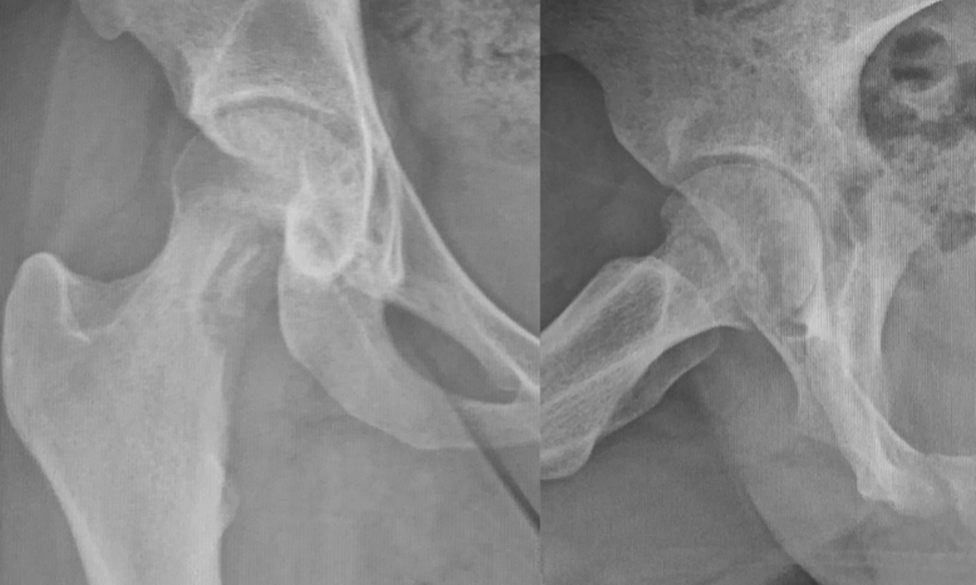
Initial x-ray demonstrating an osteolytic lesion at the femoral neck. AP on the left and lateral on the right side.

**Fig. 2:**
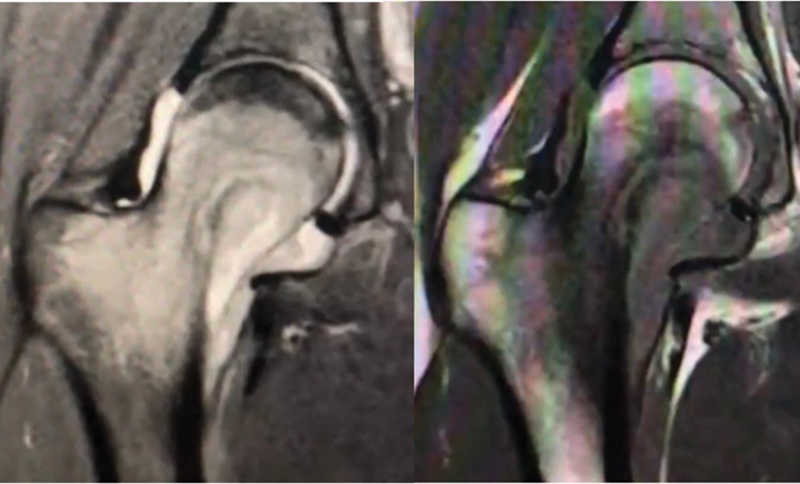
Initial Coronal MRI images, demonstrating bone marrom edema and incread intra-articular fluid.

**Fig. 3:**
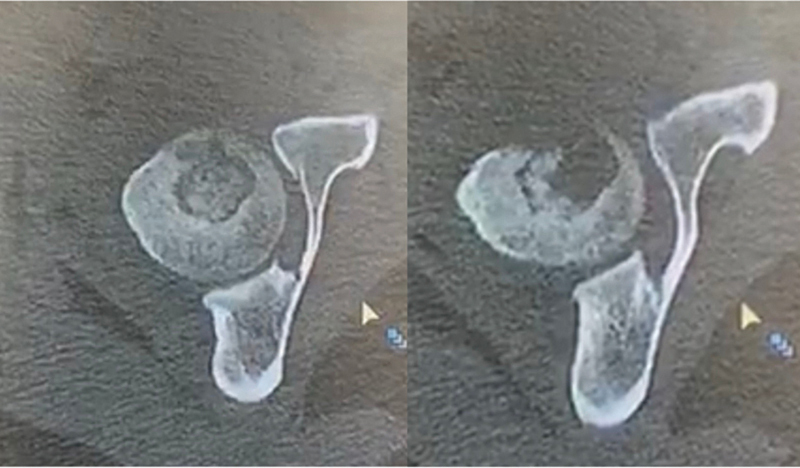
Initial axial images of the CT-scan demonstrating cortical erosion and a cavitary lesion.

A radiograph revealed an osteolytic lesion at the femoral neck ( Fig. 1 ). A magnetic resonance imaging (MRI) scan showed a hyperintense lesion and areas of necrosis within the femoral neck, in addition to an increased amount of intra-articular fluid, suggesting osteomyelitis combined with septic arthritis of the hip ( Fig. 2 ). A computed tomography scan revealed bone sequestration ( Fig. 3 ).

We performed an open surgical biopsy, collected bone and synovial tissue for culture, and drained hip arthritis. Then, we instituted antibiotic therapy for probable bacterial osteomyelitis.

Pain and fever improved. Cultures revealed no evidence of bacterial growth. The anatomopathological examination showed yeast-like structures consistent with *Cryptococcus* , leading to the diagnosis of fungal osteomyelitis.

Intravenous treatment with amphotericin B was performed for 2 weeks, resulting in complete joint pain resolution. Next, we decided to institute a clinical treatment for osteomyelitis, with oral administration of fluconazole for 6 months.

Partial load was allowed after 2 weeks and progressively increased after this period. The patient presented no hip complaints or side effects from medication, except for residual hair loss.

**Fig. 4:**
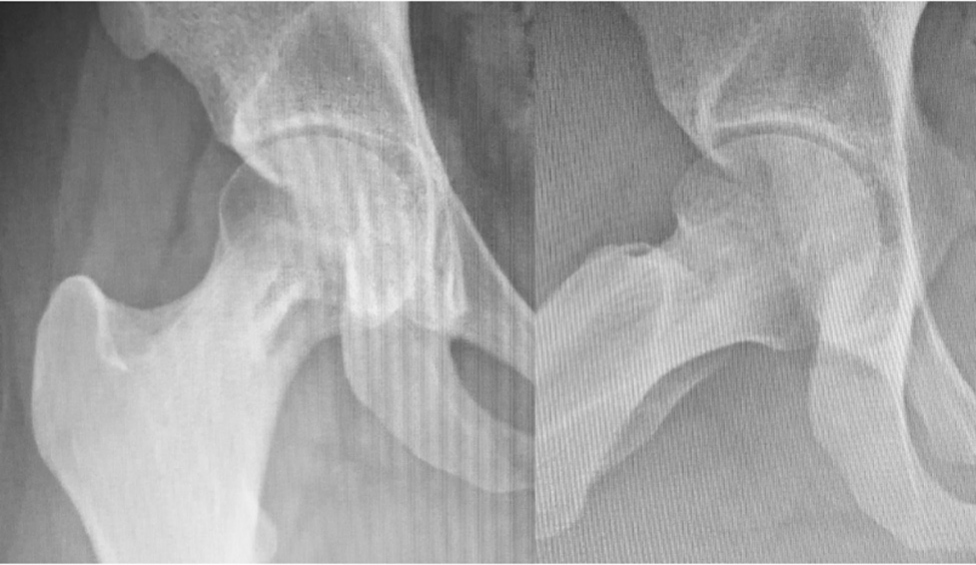
Radiograph taken 180 days after surgery demonstrating bone formation on the previous lesion.

**Fig. 5:**
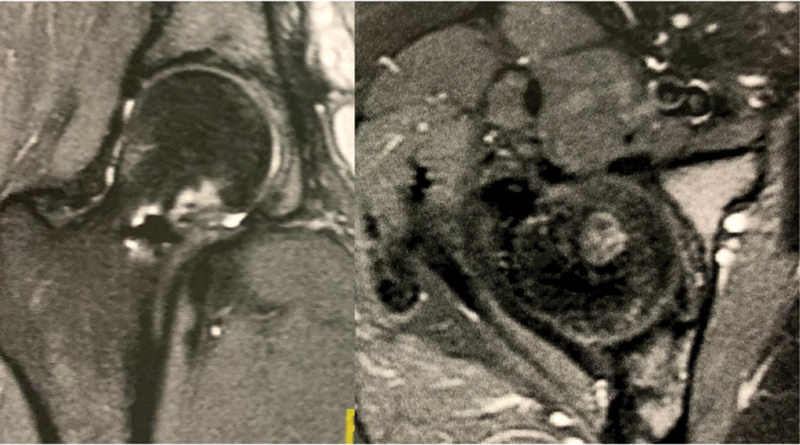
MRI 1 year after surgery, demonstrating scar tissue with bone remodeling.

Follow-up radiographs at 45, 90, and 180 days after the procedure showed progressive lesion improvement ( Fig. 4 ). Impact activities were allowed after this period. One year later, a control MRI revealed a scar from the osteomyelitis, and the patient reported no hip pain ( Fig. 5 ).

## Discussion

Most descriptions of fungal osteomyelitis cases are caused by *Candida* spp., with a higher prevalence of *Candida albicans* . The basidiomycete *Cryptococcus* is rarely reported in osteomyelitis, accounting for approximately 10% of the cases. 1 2 *Cryptococcus* infection occurs through inhalation, traumatic inoculation, or gastrointestinal tract contact; this fungus is associated with pigeon feces. 1 2 3 4 6 Osteomyelitis due to *Cryptococcus* often affects the knees and vertebras, but it may involve the elbow, ankle, wrist, and sacroiliac joints. 4 The disease is more prevalent in subjects aged 21 to 59 years old, 1 4 with no gender predisposition.

The patient's clinical history and physical examination are essential in cases of fungal osteomyelitis because the levels of inflammatory markers may be low or within the normal range. Although imaging techniques are not enough to make a correct, accurate diagnosis, the lack of bone formation and a local periosteal reaction indicate fungal pathogens. These patients present a cellular infiltration, predominantly of lymphomononuclear cells, which form non-caseating and, eventually, polymorphonuclear granulomas filled with fungi. Their synovial fluid is cloudy and viscous due to the presence of pus. 5 8

The most common management reported in the literature is to collect specimens for culture, request a microbiological analysis, drain the abscess, and debride the infected tissue. Therapy usually consists of amphotericin B administration for a short time, followed by an azole compound, such as fluconazole, for longer periods. 5 7 Although some authors perform treatment with antifungal agents alone and others do only arthrodesis, evidence suggests that clinical and surgical management promote better outcomes. 8
