# Supplementary material for: Fungal Osteomyelitis of the Hip with Septic Arthritis: Case Report
Source: Rev Bras Ortop (Sao Paulo). 2022 Feb 15;59(Suppl 1):e49–51. doi: 10.1055/s-0042-1742604 (PMC11254441; doi:10.1055/s-0042-1742604)
Supplement: Related texts

# Research article: Osteomielite fúngica do quadril associado a artrite séptica: Relato de caso

**Keywords:** artrite infecciosa, osteomielite, procedimentos ortopédicos

## Abstract

Osteomielites fúngicas, principalmente associadas à artrite séptica, são incomuns no Brasil, e por isso às vezes de difícil diagnóstico e tratamento. Relatamos um caso de uma paciente jovem, sem fator de risco imunossupressor, com o desenvolvimento de uma osteomielite e evolução para artrite séptica do quadril. O diagnóstico foi realizado após a sua drenagem cirúrgica e visualização do *Cryptococcus Neoformans* à anatomia patológica. Foi instaurado o tratamento antifúngico, com remissão total dos sintomas. Como não há consenso em relação ao tratamento de osteomielites fúngicas, descrevemos este relato de caso para atentar ortopedistas sobre a importância do diagnóstico diferencial nas artrites de quadril e a boa evolução do tratamento clínico após a drenagem e isolamento do patógeno.

## Introdução

Como resultado da inoculação direta de microrganismos durante traumas, cirurgias e/ou procedimentos como injeção ou aspiração das articulações, além da disseminação hematogênica em pacientes imunossuprimidos—fonte mais comum—a osteomielite fúngica com artrite séptica associada surge como doença incomum e de difícil abordagem clínica. Pacientes imunossuprimidos, em uso de quimioterápicos, corticosteroides, drogas intravenosas ilícitas, antibióticos de amplo espectro, infecção por vírus da imunodeficiência humana (HIV), transplante de órgãos, entre outros, são mais susceptíveis a infecções e doenças relacionadas à via hematogênica. 1 2 3 4

Esta via é normalmente correlacionada com a patogênese devido à grande vascularização das sinóvias e do tecido ósseo e por não possuir uma membrana basal que limite a entrada de microrganismos no espaço articular. 5 A infecção destes locais se dissemina, sendo definida por fatores relativos ao patógeno e ao hospedeiro.

Em geral, os quadros de artrite fúngica por *Cryptococcus neoformans* são por espécies de fungo isoladas no aparelho osteolocomotor, ainda que haja poucos casos descritos na bibliografia. 6 7 De fato, era previsto que a prevalência de artrites fúngicas aumentasse ao longo dos anos, já que os pacientes imunossuprimidos, que são os principais acometidos por doenças fúngicas sistêmicas, obtiveram aumento no número de diagnósticos. Cerca de metade dos pacientes acometidos por artrite criptocócica possuem algum fator predisponente, sendo que a monoartrite do joelho é o principal acometimento, ainda que existam casos de oligoartrite e poliartrite envolvendo outras articulações, como o cotovelo, a artiulação sacroilíaca, o tornozelo e a esternoclavicular. 8 Nos casos de infecção fúngica, o tratamento inicial consiste na limpeza cirúrgica associada ao uso de antifúngicos, sendo que um tratamento inadequado pode induzir invasão loco regional com evolução a óbito. 7

A osteomielite fúngica de quadril constitui uma ocorrência rara, fato que motivou essa descrição. Relatamos um caso raro de uma jovem diagnosticada com osteomielite fúngica no quadril direito, em colo femoral, associado à artrite séptica.

## Relato de caso

Uma paciente do sexo feminino, de 20 anos, caucasiana, relatou dor na região do quadril direito há aproximadamente 30 dias. Inicialmente, referiu dor ao andar, ao sentar-se por tempo prolongado e a ocorrência de calafrios e febre baixa (aferiu 37,5 °C) durante a noite. A paciente relatou uso ocasional de anti-inflamatório não esteroidal e contato direto com fezes de pombos em área rural, mas negou doenças associadas.

Ao exame físico, apresentava marcha claudicante, limitação da rotação interna do quadril direito em 15 graus, sinal do C, teste de flexão, adução, e rotação interna (FADURI) positivo.

Radiografia com lesão osteolítica em colo femoral ( Fig. 1 ). O exame de ressonância magnética evidenciou, em colo femoral, lesão hiperintensa, áreas de necrose no interior e aumento de líquido intra-articular, sugerindo osteomielite em combinação com artrite séptica do quadril ( Fig. 2 ). Já o exame tomográfico evidenciou sequestro ósseo ( Fig. 3 ).

**Fig. 1:** Radiografia inicial demonstrando lesão osteolítica no colo do fêmur. AP à esquerda e lateral à direita.

**Fig. 2:** Imagens de RM coronal iniciais, demonstrando edema de medula óssea e aumento do líquido intra-articular.

**Fig. 3:** Imagens axiais iniciais da tomografia computadorizada demonstrando erosão cortical e lesão cavitária.

Realizou-se biópsia cirúrgica aberta, coleta de osso e tecido sinovial para cultura, drenagem de artrite de quadril, e foi instaurada a antibioticoterapia por provável osteomielite bacteriana.

Houve evolução com melhora do quadro álgico e da febre. Nas culturas, não se evidenciou crescimento bacteriano, mas o exame anatomopatológico revelou estruturas leveduriformes que podem corresponder a *Cryptococcus* , indicando diagnóstico de osteomielite fúngica.

Foi realizado tratamento endovenoso com anfotericina B por 2 semanas, e a paciente evoluiu com melhora completa da dor articular. Assim, a decisão médica foi de tratamento clínico da osteomielite seguido por fluconazol via oral por 6 meses.

Foi liberada a carga parcial por duas semanas, com carga progressiva após esse período. A paciente evoluiu sem queixas no quadril e sem sintomas colaterais pelo uso da medicação, relatando apenas queda de cabelo residual.

Radiografias de acompanhamento aos 45, 90, e 180 dias com melhora progressiva da lesão ( Fig. 4 ), com a paciente sendo liberada para atividades de impacto após esse período. Após 1 ano, foi realizada ressonância de controle, na qual foi observado aspecto cicatricial da osteomielite, e a paciente não relatou queixa de dor no quadril ( Fig. 5 ).

**Fig. 4:** Radiografia realizada 180 dias após a cirurgia demonstrando formação óssea na lesão anterior.

**Fig. 5:** RM 1 ano após a cirurgia, demonstrando tecido cicatricial com remodelação óssea.

## Discussão

A maior parte das descrições de casos de osteomielite fúngica tem como patógeno a *Candida sp.* , sendo que a mais prevalente é a *Candida albicans* . Um microrganismo pouco relatado nos casos de osteomielite, gerando essa doença em aproximadamente 10% dos indivíduos infectados por ele, é o basidiomiceto *Cryptococcus* , 1 2 que pode infectar a população através da sua inalação, inoculação através de traumas ou até mesmo pelo trato gastroinstestinal, sendo ele associado a fezes de pombos. 1 2 3 4 6 Geralmente, os casos de osteomielite fúngica gerados por *Cryptococcus* afetam os joelhos e as vertebras, porém cita-se também o cotovelo, o tornozelo, o punho, e as articulações sacroilíacas, entre outras, 4 sendo mais prevalente em indivíduos entre 21 e 59 anos 1 4 e não tendo distinção entre homens e mulheres.

Nos casos de osteomielite fúngica, a história clínica do paciente e o exame físico são imprescindíveis, uma vez que marcadores inflamatórios podem estar pouco elevados ou em valores normais. Nesses casos, os exames de imagem não são suficientes para realizar o diagnóstico correto e preciso, mas a não formação óssea e uma reação periosteal na área são indicativos de patógenos fúngicos. Os achados nesses pacientes indicam infiltração celular predominante de linfomononucleares, formação de granulomas não-caseosos e, eventualmente, polimorfonucleares com fungo em seu interior, sendo que o líquido sinovial é descrito como turvo, viscoso, e com presença de pus. 5 8

O manejo mais comumente relatado na literatura é realizar a coleta de cultura, solicitando análise microbiológica, drenar o abcesso e debridar o tecido infectado. Em geral, administra-se anfotericina B por curto período, e um composto azólico e fluconazol por longo período. 5 7 Ainda que haja relatos de autores que realizam o tratamento apenas com antifúngicos e outros apenas a artrodese, existem evidências de que a combinação do manejo clínico com o cirúrgico promove melhores resultados. 8
